# Clinical efficacy of cognitive behavioral therapy combined with pinaverium bromide tablets on irritable bowel syndrome

**DOI:** 10.12669/pjms.39.4.6994

**Published:** 2023

**Authors:** Quanxi Liu, Yan Zhu, Wei Wang, Yongli Dong

**Affiliations:** 1 Quanxi Liu, Beijing Hospital of Integrated Traditional Chinese and Western Medicine, Hospital of Integrated Traditional Chinese and Western Medicine, Beijing University of Chinese Medicine, Department of Digestive, Beijing 100039, China; 2 Yan Zhu, Beijing Hospital of Integrated Traditional Chinese and Western Medicine, Hospital of Integrated Traditional Chinese and Western Medicine, Beijing University of Chinese Medicine, Department of Digestive, Beijing 100039, China; 3 Wei Wang, Beijing Hospital of Integrated Traditional Chinese and Western Medicine, Hospital of Integrated Traditional Chinese and Western Medicine, Beijing University of Chinese Medicine, Department of Digestive, Beijing 100039, China; 4 Yongli Dong, Wang Jing Hospital of CACMS, Scientific Research Office, Beijing 100102, China

**Keywords:** Irritable Bowel Syndrome, Pinaverium Bromide Tablet, Cognitive Behavior, Combination Therapy

## Abstract

**Objective:**

To investigate the clinical efficacy of cognitive behavioral therapy combined with pinaverium bromide tablets in admitted patients with irritable bowel syndrome (IBS).

**Methods:**

This is a retrospective study. A total of 60 patients with IBS admitted to Beijing Hospital of Integrated Traditional Chinese and Western Medicine between June 2021 and June 2022 were selected and randomly divided into two groups. Patients in the control group were treated with pinaverium bromide tablets, and those in the observation group were treated with cognitive behavior therapy combined with pinaverium bromide tablets. The improvement of clinical symptoms and quality of life before and after treatment was compared for the two groups, IBS-SSS scale and IBS-QOL scale were used to compare the improvement of clinical symptoms and quality of life between the two groups of patients before and after treatment. SAS score and SDS score were used to evaluate the psychology of the two groups. Adverse reactions occurring during the treatment were recorded, such as nausea and vomiting, dizziness and headache, etc.

**Results:**

The efficacy of the observation group was higher than that of the control group and the difference was significant (P<0.05). After treatment, the IBS-SSS score in the observation group and the control group decreased and the IBS-QOL score increased. The SDS score and SAS score in the observation group were better than those in the control group (P< 0.05). After treatment, there was no significant difference in adverse reactions between the observation group and the control group (P > 0.05).

**Conclusion:**

Cognitive behavioral therapy combined with pinaverium bromide tablets is significantly effective in the treatment of patients with IBS, which can effectively relieve symptoms such as diarrhea and abdominal pain, and reduce irritable bowel reactions.

## INTRODUCTION

Irritable bowel syndrome (IBS) is a common functional gastroenteropathy characterized by symptoms such as abdominal pain, bloating and bowel habits.^1^ There are four types of IBS, including IBS with diarrhea, IBS with constipation, IBS with mixed symptoms, and an indeterminate type, with diarrhea as the main type.^2^ The etiology and mechanism of IBS are currently unknown. Studies have shown that IBS may occur due to factors such as irregular diet, intestinal flora imbalance, gastrointestinal infection, and psychological stress.^3^,^4^ Pinaverium bromide is a commonly used gastrointestinal reliever in clinical practice for symptomatic treatment of intestinal dysfunction, gallbladder dyskinesia and intestinal spasm and can effectively relieve IBS symptoms.^5^

Pinaverium bromide monotherapy, however, has been unable to meet the clinical needs with the development of modern medicine. Cognitive behavioral therapy (CBT) is an intervention on the psychological status of patients to improve their dysfunctional emotions, such as anxiety and depression, and achieve therapeutic effects.^6^ A number of previous studies have demonstrated that CBT improves anxiety, depression, QOL, and abdominal symptoms in patients with IBS.^7^,^8^ On this basis, the present study was designed to investigate the clinical effect of CBT combined with pinaverium bromide tablets in patients with IBS to provide a clinical reference for the recovery of gastrointestinal function in patients with IBS.

## METHODS

This is a retrospective study. A total of 60 patients with IBS admitted to Beijing Hospital of Integrated Traditional Chinese and Western Medicine between Jun. 2021 and Jun. 2022 were selected and divided into the observation group (n=30) and the control group (n=30) using the simple random grouping method and random number table. No differences in general data such as sex, age, BMI and disease duration were observed between the two groups (P>0.05), which were comparable, as shown in Table-I.

**Table-I:** Comparison of general data between the two groups(n=30 each).

Group	n	Sex		Mean age (years)	Mean BMI (kg/m^2^)	Mean disease duration (months)

		Male	Female			
Observation	30	16	14	55.61±4.38	20.12±1.91	27.7±2.51
Control	30	17	13	55.78±4.46	20.91±1.81	28.1±2.62
*t*/*χ*^2^	-	0.067		0.148	1.644	0.604
*P*	-	0.795		0.882	0.106	0.548

### Ethical Approval:

The study was approved by the Institutional Ethics Committee of Beijing Hospital of Integrated Traditional Chinese and Western Medicine on November 19, 2022 (No.: ZXYEC-KT-2022-03-P04), and written informed consent was obtained from all participants.

### Inclusion criteria:

- Patients who met the diagnostic criteria of IBS.^9^
- Patients with abdominal pain and bloating.
- Patients with bowel habits: ≥25% of stools were pasty or watery, and <25% of stools were
- hard or spherical in shape.
- Patients with mucus stool.
- Patients with disease duration of > six months.
- Patients with good compliance.
- Patients with no allergy to pinaverium bromide.

### Exclusion criteria:

- Patients with tumors, severe kidney impairment, respiratory diseases, hypertension, and diabetes.
- Patients with mental illness, depression and unconsciousness.
- Patients in pregnancy or lactation.
- Patients who took gastrointestinal drugs and anti-anxiety and anti-depressants within two weeks.

Both groups of IBS patients were treated with an adjusted diet and corrected living habits after admission, including conventional interventions such as reducing intolerant diet, no cold, greasy, and spicy food, and regular diet and routine. Patients in the control group were treated with pinaverium bromide (Beijing Fuyuan Pharmaceutical Co., Ltd., batch No.: 32111005, specification: 50mg × 15 tablets/box) one tablet, three times per day, and patients in the observation group were additionally treated with CBT on the basis of the control group. Both groups of IBS patients were treated for two weeks.

*Outcome Measures:* According to the clinical efficacy evaluation criteria for IBS described previously,^10^ complete response was defined as symptoms, such as abdominal pain, diarrhea and diarrhea frequency, basically diminished and the total symptom score decreased by ≥95% after two weeks of treatment. Marked response was defined as symptoms were significantly improved and the total symptom score decreased by <95% and ≥60%. Moderate response was defined as symptoms were slightly improved and the total symptom score decreased by <60% and ≥30%. No response was defined as symptoms were not improved or even worsened, and the total symptom score decreased by <30%.

The IBS-Symptom Severity Scale(IBS-SSS) score, including abdominal pain, distension, diarrhea frequency, stool form, and anal exhaust, was compared before and after treatment for the two groups of IBS patients. Symptoms were graded into four levels according to their severity, including no symptom, mild, moderate, and severe, and cumulative scores from no symptom to severe were 0-4 points.

The IBS-quality of life scale (IBS-QOL) was used to evaluate the quality of life of the two groups of patients before and after treatment. This scale consists of 34 items, each with a 5-point response scale (not at all, slightly, moderately, quite a bit, and extremely). Cronbach’s α-coefficient was 0.71-0.89. Higher IBS-QOL scores indicated a more significant improvement in the patient’s quality of life.

Self-rating Depression Scale (SDS) and Self-rating Anxiety Scale (SAS) are commonly used to assess the emotional status of patients with IBS.^11^ The SDS scale consists of mental state, mood, physiology, general health, and physical pain, with a total score of 100, higher scores indicate more severe symptoms. The SAS scale includes 20 items to assess the levels of anxiety in patients with anxiety symptoms. Higher scores indicate more severe symptoms. Possible adverse reactions were recorded for these IBS patients in both groups during the treatment, including nausea, vomiting, dizziness, and dysphagia.

### Statistical Analysis:

All data were analyzed using SPSS 22.0 statistical software. Measurement data with normal distribution and homogeneous variance were presented as±s, two sample independent t-test was used to compare the differences between groups. Enumeration data were presented as rates, and the χ ^2^ test was used for comparison. P < 0.05 was considered statistically significant.

## RESULTS

No differences in general data, including sex, age, BMI, and disease duration were noted between the two groups (P>0.05), and the data were comparable.Table-I. The total efficacy patients with IBS in the observation group (96.67%) was higher than that in the control group (80.00%) after treatment, and the difference between the two groups was significant (P<0.05) (Table-II).

**Table-II:** Comparison of the clinical efficacy between the two groups(n=30 each, %).

Group	n	Complete response	Marked response	Moderate response	No response	Total effective rate
Observation	30	8(26.67)	12(40.00)	9(30.00)	1(3.33)	29(96.67)
Control	30	6(20.00)	11(36.67)	9(30.00)	6(20.00)	24(80.00)
*χ* ^2^	-	-	-	-	-	4.043
*P*	-	-	-	-	-	0.044

There were no significant differences in the IBS-SSS and IBS-QOL scores between the two groups before treatment (P > 0.05). The IBS-SSS score decreased significantly (P < 0.05) and the IBS-QOL score increased significantly (P < 0.05) after treatment for both groups (Table-III).

**Table-III:** Comparison of the IBS-SSS and IBS-QOL scores between the two groups (n=30 each, point, *χ̅*±*S*).

Group	n	IBS -SSS		IBS-QOL	

		Before treatment	After 2 weeks of treatment	Before treatment	After 2 weeks of treatment
Observation	30	62.41±3.41	46.11±2.37*	64.34±3.26	55.88±3.15*
Control	30	62.45±3.46	53.52±3.38*	64.53±3.29	61.17±3.42*
*T*	-	1.483	5.722	0.217	7.882
*P*	-	0.141	<0.001	0.828	<0.001

******Note:******

* P<0.05, compared with that before treatment.

There were no differences in SDS and SAS scores between the two groups before treatment (P > 0.05). SDS and SAS scores of the observation group and the control group decreased after treatment, and the SDS and SAS scores of the observation group were lower than those of the control group with statistically significant differences (P < 0.05) (Table-IV). The incidences of adverse reactions were 13.33% in the observation group and 16.67% in the control group after treatment with no significant difference between the two groups (P > 0.05) (Table-V).

**Table-IV:** Comparison of the SDS and ASA scores between the two groups (n=30 each,point,*χ̅*±*s*).

Group	n	SDS		SAS	
		Before treatment	After 2 weeks of treatment	Before treatment	After 2 weeks of treatment
Observation	30	66.33±5.12	54.21±4.37*	66.35±5.53	49.24±4.39*
Control	30	66.58±5.11	61.36±4.64*	65.63±5.62	55.34±5.12*
t	-	0.189	6.144	0.500	4.954
P	-	0.851	<0.001	0.619	<0.001

******Note:******

* P<0.05, compared with that before treatment.

**Table-V:** Comparison of adverse reactions between the two groups(n=30 each, %).

Group	n	Nausea and vomiting	Headache and dizziness	Dysphagia	Incidence
Observation	30	1(3.33)	1(3.33)	2(6.67)	4(13.33)
Control	30	2(6.67)	1(3.33)	1(3.33)	5(16.67)
χ^2^	-	-	-	-	0.131
P	-	-	-	-	0.718

## DISCUSSION

In the present study, pinaverium bromide tablets combined with CBT were used in IBS patients and the results showed that the total efficacy of patients with IBS in the observation group was 96.67%, significantly higher than that in the control group (80.0%). IBS-SSS score decreased and IBS-QOL score significantly increased after treatment for both groups, and the IBS-SSS and IBS-QOL scores of the observation group were better than those of the control group, indicating that the IBS symptoms and quality of life of these patients were effectively improved after treatment. The incidences of adverse reactions were low for the two groups after treatment with no significant differences. The reasons might include that pinaverium bromide is highly selective to intestinal smooth muscle, which can inhibit Ca^2+^ channels to prevent calcium influx into the cells.

IBS is a common digestive system disease with symptoms such as abdominal pain, diarrhea, and abdominal discomfort, and seriously affects the physical and mental health of patients owning to its long course and repeated symptoms.^12^ The pathogenesis and etiology of IBS are complicated with no definite conclusion reached.^13^ Clinical treatments for IBS include gastrointestinal spasmolytics and flora regulators with dietary regulation. Although symptoms such as abdominal pain and diarrhea may be temporarily reduced, the disease cannot be completely resolved by these treatments.^14^ Pinaverium bromide is a calcium ion antagonist, which can reduce smooth muscle contraction by preventing calcium influx into intestinal smooth muscle cells.

It is mainly used in digestive tract diseases to effectively relieve abnormal bowel movements and abdominal pain caused by digestive dysfunction.^15^ Studies have shown that psychological stress response can significantly affect intestinal sensitivity, intestinal smooth muscle contraction and secretion in IBS patients.^16^ Anxiety and depression in IBS patients are positively correlated with intestinal and visceral pain sensitivity.^17^ Assessment of the mental status of IBS patients is helpful to clarify the relationship between psychological factors and this disease. Cognitive behavioral therapy combined with pinaverium bromide tablets is significantly effective in the treatment of patients with IBS, which can effectively relieve symptoms such as diarrhea and abdominal pain, and reduce irritable bowel reactions.

It can also prevent excessive contraction of intestinal smooth muscle, improve intestinal sensitivity, and increase intestinal peristalsis, thus alleviating discomfort such as abdominal pain.^18^ The results of the present study suggested that pinaverium bromide tablets can effectively improve the gastrointestinal microenvironment and relieve the symptoms of abdominal pain and abnormal defecation, with definite efficacy on IBS and few side effects. According to Bor et al^19^, Pinaverium is superior to placebo for the treatment of IBS symptoms, irrespective of patient age or gender, study publication year, sample size.

The etiology and pathogenesis of IBS are complicated. Some scholars believe that factors such as changes in the social environment and psychological status and emotional instability may affect the progress of IBS.^20^ Patients are prone to depression and anxiety when worrying about their condition and stress increase, which may increase the sensitivity of the gastrointestinal tract, aggravate the IBS condition, and make the treatment more difficult.^21^ CBT can fight this disease by improving the patients’ negative emotions, reducing their fear of the disease and ultimately building their confidence.^22^ CBT in IBS is a targeted therapy.^23^ Studies have shown that the visceral sensitivity, gastrointestinal symptoms and mental symptoms of patients treated with CBT are significantly improved.^24^

The results of the present study showed that the SDS and SAS scores of the observation group and the control group decreased after treatment, and the scores of the observation group were significantly lower than those of the control group, which was consistent with the conclusions from previous studies. Pinaverium bromide monotherapy improved the severity of IBS symptoms to a certain extent, and the mental and psychological symptoms, IBS symptom severity and quality of life, however, were significantly improved when these IBS patients were treated with pinaverium bromide combined with CBT, indicating that CBT played a positive role in the treatment of IBS by effectively alleviating the negative emotions of IBS patients, improving their confidence in the recovery, and thus achieving the therapeutic effect of IBS. Recent studies have shown that psychological intervention is an effective treatment for IBS, especially CBT has been strictly tested in clinical trials and has shown significant and lasting symptom improvement.^25^

### Limitations of this study:

It was a retrospective descriptive study, with limited clinical data available and limited persuasive conclusions. Further intervention trials are needed in the future to confirm these results.

## CONCLUSION

CBT combined with pinaverium bromide can effectively improve IBS symptoms of abdominal pain, diarrhea frequency and stool form, increase the quality of life, relieve psychological anxiety, and ensure the safety of the clinical medication, with importance in clinical application.

### Authors’ Contributions:

**QL** and **YD:** Designed this study, prepared this manuscript, are responsible and accountable for the accuracy and integrity of the work.

**YZ:** Collected and analyzed clinical data.

**WW:** Data analysis**,** Significantly revised this manuscript.
